# miR-21 may acts as an oncomir by targeting RECK, a matrix metalloproteinase regulator, in prostate cancer

**DOI:** 10.1186/1471-2490-12-14

**Published:** 2012-05-29

**Authors:** Sabrina Thalita Reis, José Pontes-Junior, Alberto Azoubel Antunes, Marcos Francisco Dall’Oglio, Nelson Dip, Carlo Camargo Passerotti, Guilherme Ayres Rossini, Denis Reis Morais, Adriano Joao Nesrallah, Camila Piantino, Miguel Srougi, Katia R Leite

**Affiliations:** 1 Laboratory of Medical Investigation (LIM55), Urology Department, University of Sao Paulo Medical School, Sao Paulo, Brazil; 2 Uro-Oncology Group, Urology Department, University of Sao Paulo Medical School and Institute of Cancer Estate of Sao Paulo (ICESP), Sao Paulo, Brazil; 3 Urology department, Nove de Julho University, Sao Paulo, Brazil

**Keywords:** Prostate cancer, Prognosis, RECK, Micro RNA, Metaloproteinases

## Abstract

**Background:**

Prognosis of prostate cancer (PCa) is based mainly in histological aspects together with PSA serum levels that not always reflect the real aggressive potential of the neoplasia. The micro RNA (miRNA) mir-21 has been shown to regulate invasiveness in cancer through translational repression of the Metaloproteinase (MMP) inhibitor RECK. Our aim is to investigate the levels of expression of RECK and miR-21 in PCa comparing with classical prognostic factors and disease outcome and also test if RECK is a target of miR-21 in *in vitro* study using PCa cell line.

**Materials and methods:**

To determine if RECK is a target of miR-21 in prostate cancer we performed an *in vitro* assay with PCa cell line DU-145 transfected with pre-miR-21 and anti-miR-21. To determine miR-21 and RECK expression levels in PCa samples we performed quantitative real-time polymerase chain reaction (qRT-PCR).

**Results:**

The *in vitro* assays showed a decrease in expression levels of RECK after transfection with pre-miR-21, and an increase of MMP9 that is regulated by RECK compared to PCa cells treated with anti-miR-21. We defined three profiles to compare the prognostic factors. The first was characterized by miR-21 and RECK underexpression (N = 25) the second was characterized by miR-21 overexpression and RECK underexpression (N = 12), and the third was characterized by miR-21 underexpression and RECK overexpression (N = 16). From men who presented the second profile (miR-21 overexpression and RECK underexpression) 91.7% were staged pT3. For the other two groups 48.0%, and 46.7% of patients were staged pT3 (p = 0.025).

**Conclusions:**

Our results demonstrate RECK as a target of miR-21. We believe that miR-21 may be important in PCa progression through its regulation of RECK, a known regulator of tumor cell invasion.

## Background

Prostate cancer (PCa) is the most common cancer affecting males and the second leading cause of death in men in many countries, including Brazil. For 2011 903,500 new cases and 258,400 deaths are estimated worldwide [1]. Tumor staging, Gleason score and Prostatic specific antigen (PSA) serum levels are the most important prognostic factors; however, even taken together they cannot perfectly predict which patients are at risk for progression [2]. There is a variety of treatment options that ranges from active surveillance to androgen deprivation. Therefore, identification of molecular markers that could contribute to prediction of PCa behavior has been an area of active research.

MicroRNAs (miRNAs) are a class of small noncoding RNA that regulate the expression of target genes by promoting translational repression or degradation of mRNAs [3,4]. In recent years, abnormalities in miRNA expression have been identified in the progression of various cancers and consequently have been proposed as potential targets for anticancer therapies [5]. miRNAs have been shown to function as both tumor suppressors or oncogenes in various cancers [6,7]. Using high-throughput profiling of miRNA expression, miR-21 was identified as being strongly elevated in many tumors including breast, colorectal, and hepatocellular carcinoma [8-11]. However, our knowledge of the molecular mechanisms underlying the function of miR-21 in cancer generally, and prostate cancer specifically, is limited.

miR-21 regulates the expression of multiple mRNA targets associated with tumor invasiveness and microvascular proliferation. It has been shown that mir-21 regulates cell invasiveness by directly controlling the MMP inhibitor RECK (Reversion-inducing cysteine-rich protein with Kazal motifs), a key inhibitor of several MMPs. Indeed, RECK expression levels are predictive in determining prognoses in a number of common cancers; low levels of RECK are often associated with increased invasiveness and poor prognosis [12,13]. Additionally, it has been shown that inhibition of miR-21 results in reduced MMP activity leading to reduction of cell motility and invasiveness, through increased expression of RECK [14].

In this study, we will investigate if RECK is a target of miR-21 in prostate cancer cell line DU-145 in *in vitro* assays. Also we will evaluate the expression levels of RECK, MMP9 controlled by RECK and miR-21 in PCa tissue comparing with Gleason score, pathological stage, pre-operatory PSA serum levels and the outcome of patients with localized PCa treated with radical prostatectomy.

## Methods

### Patients and tissue samples

The demographic and clinical characteristics of the 53 patients included in the study are exposed in Table1. After surgery, their PSA was measured every six months for the first five years and then once annually after that. Patients who underwent adjuvant or neoadjuvant treatment were excluded from this study. All subjects provided informed consent for study participation and to allow their biological samples to be analyzed genetically. The study was approved by the Institutional Board of Ethics (CAPPesq, Hospital das Clpinicas da Faculdade de Medicina da Universidade de São Paulo, no. 0453/08).

**Table 1:** Demographic and clinical characteristics of 53 patients included in the study submitted to radical prostatectomy to treat prostate cancer

Age (years) Mean	65.4
Min - Max	50 - 79
PSA (ng/ml) Mean	11.0
Min - Max	2.0 – 37.0
<10 n (%)	27 (50.1)
≥10 n (%)	26 (49.9)
Stage	
pT2 n (%)	22 (42.3)
pT3 n (%)	30 (57.7)
Gleason Score	
< 7 n (%)	22 (41.5)
≥ 7 n (%)	31 (58.5)

We analyzed miR-21, RECK and MMP9 expression levels in fresh-frozen tumor specimens randomly selected from our tumor bank from patients with clinically localized PCa who underwent radical prostatectomy by a single surgeon between 1993 and 2007 using quantitative real-time polymerase chain reaction (qRT-PCR) using TaqMan primers. The control group consisted of tissue specimens from 11 patients with benign prostatic hyperplasia (BPH) who presented lower urinary tract symptoms and underwent retropubic prostatic resection (mean age 64±6.0 years).

We found three different profiles characterized as follows: [1] miR-21 and RECK underexpression (n = 25) [2] miR-21 overexpression and RECK underexpression or [3] miR-21 underexpression and RECK overexpression (n = 16).

Then the three profile were correlated with Gleason score, pathological stage (TNM 2010) and pre-operative serum PSA levels (ng/mL). For this analysis, pathological stage was defined as organ-confined (pT2) or non-organ-confined (pT3). Gleason score was classified as low grade (Gleason score ≤6) or high grade (Gleason score ≥7). Preoperative PSA was divided as ≥10 ng/mL or <10 ng/mL. Additionally, we analyzed miR-21 and RECK expression levels and their relation to biochemical recurrence, which was defined as PSA levels >0.4 ng/mL, in a mean follow-up time of 66.2 months for all patients.

### RNA and miRNA isolation and cDNA synthesis

All tumor samples were obtained from surgical specimens and immediately frozen at −170°C in liquid nitrogen. A section of the frozen fragment was stained with hematoxylin and eosin to verify the presence of tumor in at least 75% of the fragment in PCa patients and if there was no tumor in control BPH patients. Total RNA was isolated from tissue samples using a RNAaqueous Kit (Applied Biosystems, CA, USA) and miRNA was isolated with a mirVANA Kit (Applied Biosystems, CA, USA) according to the manufacturer’s instructions. RNA and miRNA concentration was determined by 260/280 nm absorbance using a Nanodrop ND-1000 spectrophotometer (Thermo Scientific).

cDNA from total RNA was generated using a High Capacity cDNA Reverse Transcription Kit (Applied Biosystems, CA, USA). Reactions were incubated at 25°C for 10 min, followed by 37°C for 120 min and 85°C for 5 min. cDNA from miRNAs was generated using a TaqMan® miRNA Reverse Transcription kit (Applied Biosystems, Foster City, CA). Reactions were incubated at 16°C for 30 min, 42°C for 30 min and 85°C for 30 min. All cDNAs were stored at −20°C until further use.

### Quantitative real-time PCR of miR21, RECK and MMP9

Expression levels of the miR-21 and RECK were analyzed by qRT-PCR using the ABI 7500 Fast Real-Time PCR System (Applied Biosystems). Target sequences were amplified in a 10-μl reaction containing 5 μl of TaqMan Universal PCR Master Mix, 0.5 μl of TaqMan Gene Expression Assays for RECK and 0.5 μl of TaqMan miRNA expression assays for miR-21, 1 μl of cDNA and 3.5 μl of DNase-free water. The PCR cycling conditions were 2 minutes at 50°C, 10 minutes at 95°C and then 40 cycles of 15 seconds at 95°C and 1 min at 60°C. Betha-2 microglobulin was used as the endogenous control in the analysis of RECK expression. RNU43 was used as the endogenous control for miR-21 (Table2).

**Table 2:** Primers utilized

**Gene symbol**	**Assays ID**
**Mir21**	002438
**RNU43**	001095
**RECK**	Hs01019179_m1
**MMP9**	Hs00957562_m1
**B2M**	Hs99999907_m1

We used the ΔΔCT method to calculate the relative expression of the RECK and miR-21 using the formula ΔΔCT = (CT target gene, PC sample - CT endogenous control, PC sample) – (CT target gene, BPH sample - CT endogenous control, BPH sample). The fold change in gene expression was calculated as 2^-ΔΔCT^.

### Cell lines

Human prostate cancer DU145 was purchased from ATCC (American Type Culture Collection, Manassas, USA). The cell lines were cultured in McCoy's medium supplemented with 20% (v/v) heat-inactivated fetal bovine serum (Sigma, St.Louis, MO, USA) under an atmosphere of 5% CO2 at 37°C. At the time of confluence, adherent cells were subcultured after detachment using trypsin/EDTA (0.25% trypsin- 1.0 mM ethylenediaminetetraacetate).

### Cell transfection

The transfection of Pre-miR-21 and its antagomir anti-miR-21 via NeoFX was performed by following the manufacturer’s manual. Briefly, 2.5 μl de Pre e anti – miR-21 was mixed with 50 μl OptiMEM, and 1.5 μl of NeoFX was mixed with 50 μl OptiMEM. This dilutions were mixture and then, were incubated at room temperature for 10 min. Untreated cells and cells transfected by scrambled microRNA precursor with no target were negative controls in our work.

### Quantification of miR-21 and RECK, MMP9 mRNA expression in DU145 cells by quantitative real time PCR

Twenty-four hours post transfection, the cells were washed with 1xPBS and then RNA and miRNA were extracted with mirVANA Kit (ambion), cDNA from total RNA was generated using a High Capacity cDNA Reverse Transcription Kit (Applied Biosystems, CA, USA) and from miRNAs was generated using a TaqMan® miRNA Reverse Transcription kit (Applied Biosystems, Foster City, CA). The qRT-PCR amplification of cDNA was performed using TaqMan MicroRNA assay for miR-21, and TaqMan gene expression assay for RECK and MMP-9.

### Statistical analysis

Qualitative variables were expressed as numbers and percentages. To compare the clinical characteristics of patients with PCa, we used the Mann–Whitney or T Student, Chi-squared and Fisher exact tests. Statistical analysis was performed using SPSS 15.0 for Windows using a significance of p ≤0.05.

## Results

**Figure 1:**
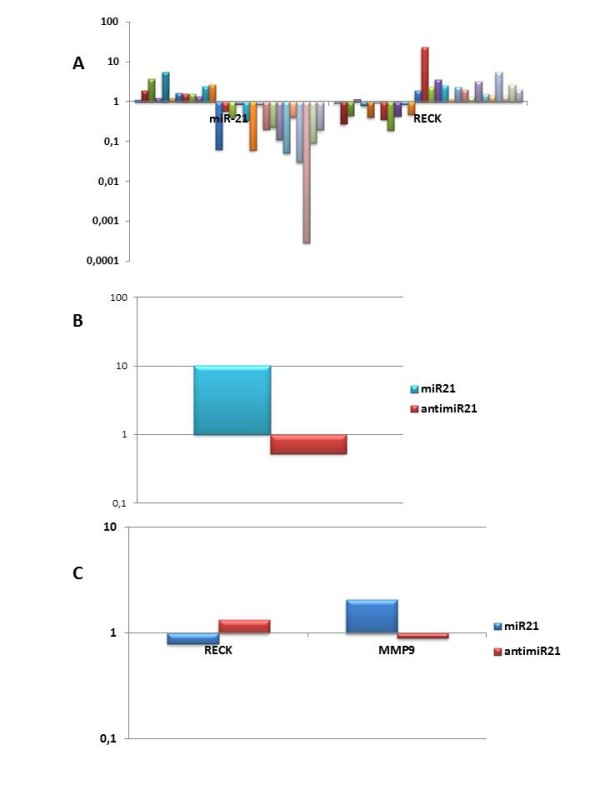
**(A) Graphical representation of the expression levels of miR-21 and RECK in 27 patients with PCa compared with BPH which included in two profiles:** miR-21 overexpressed - RECK underexpressed, and miR-21 underexpressed - RECK overexpressed. (B) Graphical representation of the expression levels of miR-21 in PCa cell treated with pre-miR-21 e anti-miR-21. (C) Graphical representation of the expression levels of RECK and MMP9 in PCa cell treated with pre-miR-21 e anti-miR-21. Fold change in expression was calculated using the 2^-ΔΔCT^ method.

Analysis of 53 patients with PCa showed that miR-21 was overexpressed in 42.8% (median 1.64) and RECK in 57.2% (median 2.2) of malignant prostatic tissue samples, relative to control BPH samples (Figure 1.A). miR-21 and RECK expression data and their correlation with prognostic parameters are presented in Table3. Since RECK is a target of miR-21, we defined three profiles for analysis. The first profile contained samples in which miR-21 and RECK were underexpressed, 25 cases (47.1%). The second profile was composed of samples in which miR-21 was overexpressed and RECK underexpressed, 12 cases (22.6%), and the third profile was composed of samples in which miR-21 was underexpressed and RECK overexpressed, 16 cases (30.2%). Comparing the pathological stage between the three groups we observed that that 91.7% of patients were staged pT3 when miR-21 was overexpressed and RECK underexpressed. On the contrary, when miR-21 was underexpressed and RECK overexpressed only 46.7% of patients were staged pT3, and when both were underexpressed only 48.0% of patients were staged pT3 (p = 0.025) (Table4).

**Table 3:** miR-21 and RECK expression data in the malignant prostatic tissue according to Gleason score, Pathological stage, PSA value and Biochemical Recurrence

	**miR-21**	**RECK**
**Gleason Score**		
< 7 (n = 22)	0.63	0.88
≥ 7 (n = 31)	0.81	2.10
p-value	0.545	0.214
**Pathological Stage**		
pT2 (n = 22)	0,36	1.18
pT3 (n = 30)	1,01	1.85
p-value	**0.009**	0.470
**PSA value**		
<10 (n = 27)	0.68	0.94
≥10 (n = 26)	0.80	2.18
p-value	0.701	0.390
**Biochemical Recurrence**		
W/n (n = 27)	0.52	1.05
With (n = 20)	1.12	2.61
p-value	**0.051**	0.366

Fold change in gene expression was calculated using the ΔΔCT method (QRel = 2-ΔΔCT).

**Table 4:** Relative expression of miR-21 and RECK in the malignant prostatic tissue in three profiles

	**↓miR-21 and** **↓RECK**	**↑miR-21 and** **↓RECK**	**↓miR-21 and** **↑RECK**	**P value**
**Gleason Score**				
< 7	56.0%	33.3%	31.3%	0.215
≥ 7	44.0%	67.7%	68.7%	
**Pathological Stage**				
pT2	52.0%	8.3%	53.3%	**0.025**
pT3	48.0%	91.7%	46.7%	
**PSA value**				
<10	60.0%	50.0%	37.5%	0.371
≥10	40.0%	50.0%	62.5%	
**Biochemical Recurrence**				
No	75.0%	36.4%	50.0%	**0.087**
Yes	25.0%	63.6%	50.0%	

miR-21 and RECK underexpressed, miR-21 overexpressed and RECK underexpressed, and miR-21 underexpressed and RECK overexpressed, correlated either with Gleason score, pathological stage, PSA serum value or biochemical recurrence. Fold change in gene expression was calculated using the ΔΔCT method (QRel = 2-ΔΔCT).

Additionally, there was a marginal statistical difference between the three profiles when we examined biochemical recurrence. From patients presenting miR-21 overexpression and RECK underexpression, 63.6% had biochemical recurrence compared with 50.0% and 25.0% of patients in the others profiles (p = 0.087) (Table4).There were no differences in the three groups considering Gleason score or PSA pre-operatory serum levels.

The expression level of MMP9 having as a control BPH tissue was 5.32 times when miR21 was overexpressed and RECK underexpressed, and 5.48 times when both miR21 and RECK were underexpressed. When miR21 was underexpressed and RECK overexpressed MMP9 was 3.1 times.

The in vitro assays after 24 h of DU145 exposition to pre-miR21 and anti-miR-21, showed a 20 times increase in miR21 expression, confirming the success of transfection (Figure 1.B). The supposed target gene RECK showed two times reduction in expression when exposed to pre-miR-21. Also, MMP9, regulated by RECK had doubled its expression levels after exposition to pre-miR21 (Figure 1.C).

## Discussion

To understand the role of any miRNA in disease such as cancer, the mRNA targets of that miRNA must be identified. Each miRNA has hundreds of targets and each mRNA is target for different miRNAs. miR-21 plays a role in the regulation of cell proliferation, apoptosis and tumor invasiveness by targeting PTEN, PDCD4, and RECK [15], and has been shown to be involved in gliomas carcinogenesis process. This is the first study to explore the role of miR-21 controlling RECK in PCa cell lines and PCa specimens. In PCa cell line DU-145 miR-21 showed that directly inhibits RECK, a tumor suppressor gene that is involved in the control of MMP9. Studying 53 PCa patient samples, 22.6% exhibited miR-21 overexpression and RECK underexpression associated with non-organ confined tumors (pT3). We therefore believe that miR-21 may be playing a role in a subset of prostate cancers through regulation of RECK expression levels.

The same phenomenon has been observed in other types of human cancers. Liu et al. showed that inhibition of miR-21 lead to a decrease in invasiveness in hepatocellular carcinoma [15]. Inhibition of miR-21 leads to increasing levels of RECK, a membrane-anchored glycoprotein that inhibits tumor cell invasion by regulating MMP-2, MMP-9 and MT1-MMP [16]. In tumors in which RECK is absent or diminished, MMPs will be highly active, facilitating tumor promotion and progression. In our study, we showed in PCa cell line transfected with pre-miR-21, a decreased in the levels of RECK mRNA and consequently an increased in the level of MMP9.

Recently, Zhang et al. have described the similar results in gastric cancer [17]. Cancers which overexpressed miR-21 and had *Helicobacter pylori* infection had a higher capacity for invasion and migration of tumor cells. Here, we have demonstrated a similar association in PCa where 91.7% of tumors presenting overexpression of miR-21 were staged pT3.

The miR-21 is one of the most commonly implicated miRNAs in cancer. Its expression is highly upregulated in a variety of solid tumors including gliomas, breast, lung and pancreas carcinomas [18,19]. Elevated miR-21 expression has been causally linked to increase in cell proliferation, reduction in apoptosis, and migration of several cancer cell lines [20]. However, the molecular mechanisms mediating miR-21 function in cancer generally, and PCa specifically, are poorly understood. MiR-21 regulates RECK, which we have observed can function as a tumor suppressor gene in PCa (unpublished data). RECK decreases the amount of active MMP-2 and MMP-9 in conditioned medium and inhibits metastatic activity in vitro [21] and in vivo [22] through modulation of these MMPs, which are known to be involved in cancer progression [23].

Our data show a tendency in association between miR-21 overexpression and biochemical recurrence. When miR-21 was overexpressed and RECK was underexpressed 63.4% of patients suffered recurrence with a mean follow up of 60 months (p = 0.087). We may hypothesize that this specific profile is unfavorable for prostate adenocarcinoma, and a higher number of patients should be studied to confirm this preliminary finding.

In this first study we identify a possible role of miR-21 in the behavior of prostate cancer promoting a decay in the levels of RECK mRNA allowing the overexpression of MMP9. Interesting would be to prove also the decay in protein levels using westerbloting, and this is a theme of our next study.

## Conclusions

In conclusion, we believe that miR-21 may be important in PCa progression through its regulation of RECK, a known regulator of tumor cell invasion. Experimental studies must be performed in order to show the precise role of miR-21 and MMP and its regulators, specially RECK in prostate carcinogenesis. Consequently, suppression of miR-21 would be considered as a potential therapeutic tool in the treatment of the neoplasia.

## Abbreviations

BPH, Benign prostatic hyperplasia; cDNA, Complementary deoxyribonucleic acid; ECM, Extracellular matrix; MMP, Matrix metalloproteinase; PCa, Prostate cancer; PSA, Prostate-specific antigen; qRT-PCR, Quantitative real-time polymerase chain reaction; RECK, Reversion-inducing cysteine-rich protein with Kazal motif; RNA, Ribonucleic acid; TIMP-1, Tissue inhibitor of metalloproteinases 1.

## Competing interests

The authors declare that they have no competing interests

## Authors’ contributions

All authors read and approved the final manuscript.-conception and design: STR, KRL -acquisition of patients and data: MFD, JPJr, AAA, AJN- Drafting of the manuscript: STR, KRL- molecular genetic studies: STR, DRM, ND, GAR- Administration support: CP, DRM- Statistical Analysis: STR- critical revision and important intellectual content: KRL, MFD, AAA, CCP- supervision: MS.

## Pre-publication history

The pre-publication history for this paper can be accessed here:

http://www.biomedcentral.com/1471-2490/12/14/prepub
